# Association of clinicopathologic variables and patient preference with the choice of surgical treatment for early-stage breast cancer: A registry-based study

**DOI:** 10.1016/j.breast.2023.103614

**Published:** 2023-11-30

**Authors:** Emma Söderberg, Fredrik Wärnberg, Anna-Karin Wennstig, Greger Nilsson, Hans Garmo, Lars Holmberg, Carl Blomqvist, Malin Sund, Charlotta Wadsten

**Affiliations:** a Department of Surgery, Sundsvall Hospital, Sundsvall, Sweden; b Department of Surgical and perioperative Sciences/Surgery, Umeå University, Umeå, Sweden; c Department of Surgery, Institute of Clinical Sciences, Sahlgrenska Academy at University of Gothenburg, Gothenburg, Sweden; d Department of Oncology, Sundsvall Hospital, Sundsvall, Sweden; e Department of Immunology, Genetics and Pathology, Section of Experimental and Clinical Oncology, Uppsala University, University Hospital, Uppsala, Sweden; f Department of Oncology, Gävle Hospital, Gävle, Sweden; g Department of Oncology, Visby Hospital, Visby, Sweden; h Department of Surgical Sciences, Uppsala University, Uppsala, Sweden; i Translational Oncology & Urology Research (TOUR), School of Cancer and Pharmaceutical Sciences, King's College London, London, United Kingdom; j Department of Oncology, Helsinki University Hospital, Helsinki, Finland; k Department of Surgery, University of Helsinki and Helsinki University Hospital, Helsinki, Finland

**Keywords:** Breast cancer, Surgical treatment, Mastectomy, Breast conserving surgery

## Abstract

**Introduction:**

Observational studies suggest that breast conserving surgery (BCS) and radiotherapy (RT) offers superior survival compared to mastectomy. The aim was to compare patient and tumour characteristics in women with invasive breast cancer ≤30 mm treated with either BCS or mastectomy, and to explore the underlying reason for choosing mastectomy.

**Methods:**

Women registered with breast cancer ≤30 mm and ≤4 positive axillary lymph nodes in the Swedish National Breast Cancer Register 2013–2016 were included. Logistic regression analyses were performed to assess the association of tumour and patient characteristics with receiving a mastectomy vs. BCS.

**Results:**

Of 1860 breast cancers in 1825 women, 1346 were treated by BCS and 514 by mastectomy. Adjuvant RT was given to 1309 women (97.1 %) after BCS and 146 (27.6 %) after mastectomy. Variables associated with receiving a mastectomy vs. BCS included clinical detection (Odds Ratio (OR) 4.15 (95 % Confidence Interval (CI) 3.35–5.14)) and clinical stage (T2 vs. T1 (OR 3.68 (95 % CI 2.90–4.68)), N1 vs. N0 (OR 2.02 (95 % CI 1.38–2.96)). Women receiving mastectomy more often had oestrogen receptor negative, HER2 positive tumours of higher histological grade. The most common reported reason for mastectomy was large or multifocal tumours (53.5 %), followed by patient preference (34.5 %).

**Conclusion:**

Choice of surgery is strongly associated with key prognostic factors among women undergoing BCS with RT compared to mastectomy. Failure to control for all relevant confounders may bias results in outcome studies in favour of BCS.

## Highlights

• Results in observational studies comparing breast conservation to mastectomy may be influenced by a selection bias.
• Choice of surgery is strongly associated with key prognostic factors.
• Failure to control for relevant confounders may bias results in outcome studies in favour of breast conserving surgery.

## 1 Introduction

In recent years, a number of observational studies suggest that breast conserving surgery (BCS) offers superior survival compared to mastectomy for early-stage breast cancer (BC) [[1], [2], [3], [4], [5]]. In these studies, surgical treatment has not been randomized, but selected based on patient and tumour characteristics, patient or surgeon preferences, and other unknown factors that are difficult to operationalize in statistical analysis.

Mastectomy and BCS with adjuvant whole breast radiotherapy (RT) have in earlier large randomized studies been shown oncologically equivalent in terms of survival [6,7]. A meta-analysis of seven randomized studies including 3100 patients showed no difference in 10-year survival when comparing mastectomy with BCS plus RT [8]. The reason for the possible additional benefit of BCS seen in more recent observational studies is unclear. Management of BC has evolved since the large randomized trials conducted in the 80s, with improvement in surgical techniques and adjuvant treatments. There has been an increased focus on achieving clear margins when performing BCS [8]. Moreover, RT techniques have changed significantly with improved identification of target organs, while minimizing the toxicity of normal tissue [9,10]. Differences between mastectomy and BCS in outcome in these observational studies might however be influenced by a selection bias that is not fully statistically adjusted since the selection factors are not recorded or even entirely understood. Two large studies using propensity score-mathched analyses did in fact not show any survival benefit of BCS and RT compared to mastectomy [11,12].Choice of surgical treatment is influenced by multiple preoperative factors such as radiological tumour size, presence of multiple tumour foci, or widespread malignant calcifications in the breast [13]. Various medical conditions where RT is considered unsuitable also affect the recommendation for surgical treatment and may be difficult to adjust for in observational studies.

The aim of the present study was to compare patient and tumour characteristics in women with a postoperative invasive breast tumour ≤30 mm treated with a mastectomy as opposed to BCS in a population-based cohort and to explore the underlying reasons that were prospectively reported for choosing mastectomy.

## 2 Methods

### 2.1 The register

The study was a retrospective analysis of prospectively collected data from the Swedish National Breast Cancer Register (NBCR). The register was initiated in 2007, reaching national coverage in 2008. All newly diagnosed cases of BC are included with a documented coverage reported to be 99.9 % [14]. In the Northern Healthcare Region, approximately 800 patients are diagnosed with BC annually in a source population of about 900,000. Geographically, the Northern Region covers approximately 240,000 square kilometers, or 51 % of Sweden's total area, and is thus characterized by long distances between healthcare facilities. In this region, a unique register variable specifically for women treated with a mastectomy was added between 2013 and 2016, where the reason for choosing mastectomy as opposed to BCS was stated. The reasons for mastectomy were categorized as follows:

• Age below 40 years.
• Large tumour size relative to breast volume.
• Multifocal or multicentric BC.
• Inflammatory BC treated with neoadjuvant therapy.
• RT considered contraindicated.
• The patient's choice.

Registration of more than one reason for choosing mastectomy was permitted and in cases where multiple reasons were registered, the different reasons were ranked and only what was considered as the main reason was selected for the present analysis. Large tumour size relative to breast volume and multifocal BC were both considered as main reasons.

### 2.2 Material

Information regarding patient and tumour characteristics, surgery, and planned locoregional RT in women surgically treated for early-stage invasive BC ≤ 30 mm and with ≤4 positive lymph nodes between 2013 and 2016 was obtained. The tumour size up to 30 mm criterion was chosen because it should allow for both surgical approaches, either BCS or mastectomy. The NBCR includes both the clinical T-stage according to the eighth edition of the American Joint Committee on Cancer (AJCC) staging manual and the largest invasive tumour in mm, the number of invasive tumour foci, and the total extent of all invasive and *in situ* foci in the surgical specimen from the postoperative histopathology report after final surgery. According to AJCC, T1 tumours are <20 mm and T2 tumours are between 21 and 50 mm. N0 represents no regional lymph node metastasis and N1 refers to metastatic movable ipsilateral axillary lymph node(s). Patients treated with neoadjuvant therapy for BC were excluded. Women with bilateral BC, either synchronously or diagnosed within the studied time period were noted as two separate occurrences of decision-making. For women who underwent mastectomy, including those who underwent mastectomy as the final surgical procedure after an initial BCS attempt, the reported reason for mastectomy was obtained.

### 2.3 Statistics

Categorical data are presented as numbers with their percentages and continuous variables with their range. The chi-square test was used in statistical comparisons between categorical variables, while *t*-test was used for continuous variables. Statistical significance was set at p < 0.05. Logistic regression analyses were performed to assess the association of tumour and patient characteristics with receiving a mastectomy vs. BCS. Results are shown as odds ratios (OR) with 95 % confidence intervals (CI). Histopathological results of surgical specimens after mastectomy and BCS were compared by logistic regression analyses.

SPSS version 29 was used for the analyses (IBM SPSS Statistics V29.0 (IBM Corp., Armonk, NY, USA)).

### 2.4 Ethical considerations

The study was performed after ethical approval (EPM Dnr:2019–04916) and conducted in accordance to the principles of the Declaration of Helsinki.

Results were reported according to the STROBE-criteria for cohort studies.

## 3 Results

### 3.1 Patient characteristics

**Fig. 1:**
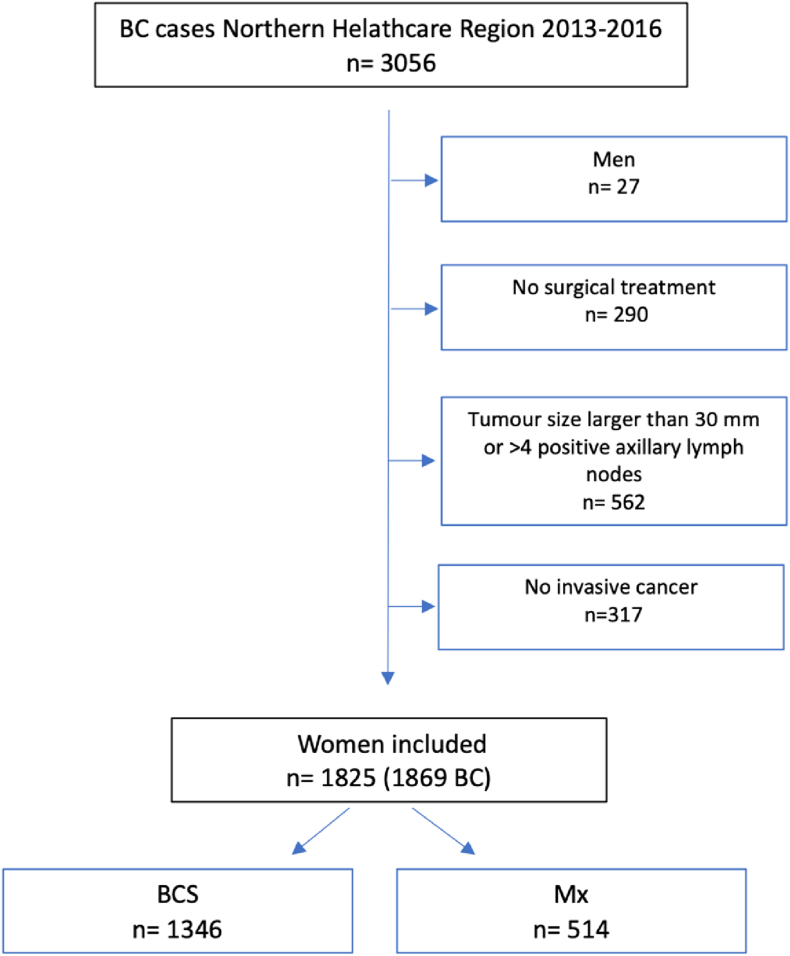
Flowchart of BC included. BC = breast cancer, BCS = breast conserving surgery, and Mx = mastectomy.

A total of 3056 BCE were registered in the Northern healthcare region between 2013 and 2016. After excluding men (n = 27), women not receiving surgical treatment (n = 290), tumours registered >30 mm or with >4 positive axillary lymph nodes (n = 562), and women with tumours without invasive components (n = 317), a total of 1860 BCE in 1825 women remained for the analysis, presented in Fig. 1. Overall, 1346 (72.6 %) women received BCS and 514 (27.4 %) women were treated with mastectomy. In the mastectomy group, 62 women were initially treated by BCS but underwent re-operation with conversion to mastectomy. In 46 women, an immediate implant-based reconstruction was performed.

Adjuvant RT to the breast or chest wall with or without ipsilateral regional lymph nodes after surgery was given to 1309 women (97.1 %) in the BCS group and 146 (27.6 %) in the mastectomy group, respectively (data not shown in table).

### 3.2 BCS versus mastectomy

Table 1 presents baseline clinical characteristics by type of surgery and the ORs for having a mastectomy vs. BCS for patient and tumour related variables. Median age was 64 years in the BCS group and 68 years in the mastectomy group. Mastectomy was more common in women younger than 40 years or older than 70 years.

**Table 1:** Preoperative variables associated with increased likelihood of receiving a mastectomy vs. BCS for women with breast cancer ≤30 mm.

	All n = 1860 (%)	BCS n = 1346 (%)	Mastectomy n = 514 (%)	Odds Ratio for having a mastectomy (95 % CI)
**Age**				
Median (range)	65 (23–93)	64 (34–89)	68 (23–93)	1.03 (1.02–1.04)
≤40 years	55 (3.0)	25 (1.9)	30 (5.8)	5.55 (3.16–9.74)
41–50 years	203 (10.9)	146 (10.8)	57 (11.1)	1.71 (1.18–2.46)
51–60 years	372 (20.0)	303 (22.5)	69 (13.4)	1.05(0.76–1.45)
61–70 years	675 (36.3)	550 (40.9)	125 (24.3)	1.0 (ref)
>70 years	555 (29.8)	322 (23.9)	233 (45.3)	3.25 (2.51–4.21)
**Detection mode**				
Screening	1121 (60.3)	937 (69.6)	184 (35.8)	1.0 (ref)
Clinical	735 (39.5)	405 (30.1)	330 (64.2)	4.15 (3.35–5.14)
Missing	4 (0.2)	4 (0.3)	0 (0.0)	n.a.
**Side**				
Right	924 (49.7)	667 (49.6)	257 (50.0)	1.0 (ref)
Left	936 (50.3)	679 (50.4)	257 (50.0)	0.98 (0.80–1.20)
**T-Stage (Clin)**				
T1	1499 (80.6)	1169 (86.8)	330 (64.2)	1.0 (ref)
T2	361 (19.4)	177 (13.2)	184 (35.8)	3.68 (2.90–4.68)
**N-stage (Clin)**				
N0	1742 (93.7)	1278 (94.9)	464 (90.3)	1.0 (ref)
N1	118 (6.3)	68 (5.1)	50 (9.7)	2.02 (1.38–2.96)

BCS = breast conserving surgery, CI = confidence interval, T-stage = Tumour stage, Clin = clinical, and N-stage = Nodal stage.

Women with clinically detected tumours as opposed to screening detected were more likely to be treated with a mastectomy (OR 4.15 (95 % CI 3.35–5.14)), as were women with a more advanced clinical stage, T2 vs. T1 (OR 3.68 (95 % CI 2.90–4.68)) and N1 vs. N0 (OR 2.02 (95 % CI 1.38–2.96)).

**Fig. 2:**
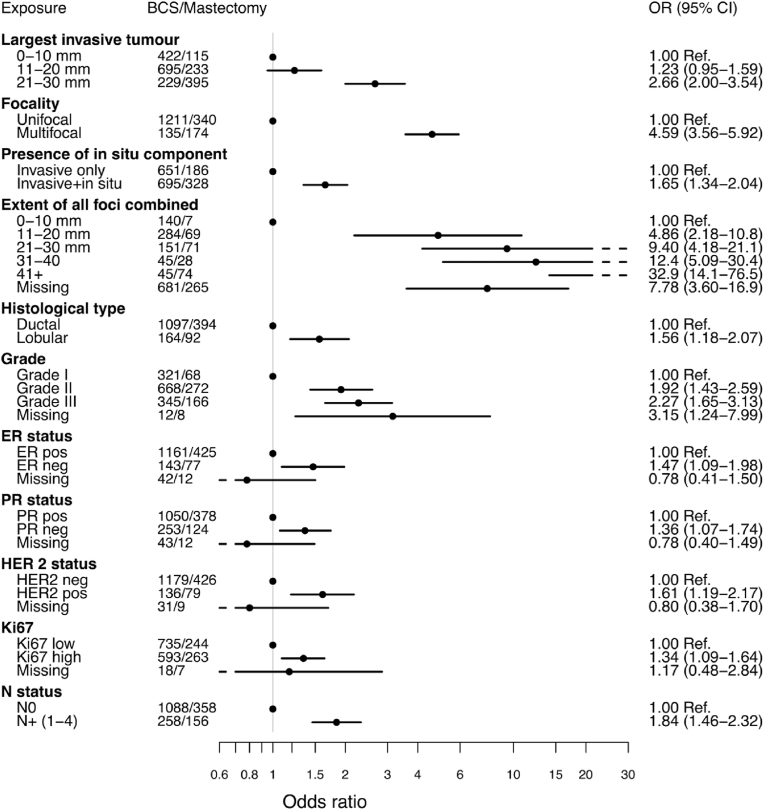
Forest plot Odds ratio for having a mastectomy vs. BCS dependent on different clinicopathological variables Distribution of reported reasons for choosing mastectomy.

Histopathological features by type of surgery are presented in Fig. 2 and Table S1. Microscopically, tumours in the mastectomy group were larger and more often multifocal (OR 4.59 (95 % CI 3.56–5.92)). In the group of women who underwent mastectomy after initial BCS, 54.8 % had multifocal tumours (Table S1).

Tumours in the mastectomy group were more often of lobular histology (OR 1.56 (95 % CI 1.18–2.07)) and of higher Nottingham Histologic Grade (NHG), NHG III vs. NHG I (OR 2.27 (95 % CI 1.65–3.13)). Immunohistochemistry showed more hormone receptor negative, and HER2 positive tumours in the mastectomy group compared to women treated with BCS.

The reason for choosing mastectomy was registered in 431 of 514 women (83.8 %), shown in Table 2. Five women were registered with more than one reason. One woman was reported younger than 40 years old with multifocal BC, one woman with a combination of large tumour size relative to breast volume and multifocal BC, and one woman had multifocal BC in combination with preferring mastectomy by her own choice. These three women were classified as multifocal BC. Two women were reported to have large tumour size relative to breast volume but also preferring mastectomy by their own choice, and these were classified as large tumour size as the main reason (not shown in table).

**Table 2:** Distribution of registered reasons for receiving a mastectomy in women with breast cancer ≤30 mm in the Northern healthcare region.

	No. of patients (N = 431)	%
Age <40 years	6	1.4
Large tumour size/extent relative to breast volume	140	32.5
Multifocality	95	21.0
RT contraindicated due to comorbidity	41	9.5
Patient's own choice	149	34.5

NO. = Number, RT = radiotherapy.

Age distribution and clinical variables between women with or without registered reason for mastectomy were similar with the exception that a larger proportion of women with registered reason had BC detected by screening (Table S2). For about two-thirds of the women, mastectomy was reported to be recommended for tumour- or patient-related medical reasons. For 9.5 % of the women, RT was considered unsuitable due to comorbidity. For the remaining women it was reported to be the woman's own choice (Table 3). Six women with insufficient surgical margins were reported to have chosen mastectomy instead of a local re-excision. A separate analysis, excluding the women who underwent a mastectomy after initial BCS did not alter overall results (Table S1).

**Table 3:** Comparison between women treated by breast conserving surgery and the subgroup of women receiving mastectomy by their own preference.

	BCS n = 1346 (%)	Mastectomy by own choice n = 149 (%)	p
***Age***			
Median (range)	64(34–89)	72(34–90)	<0.01
≤40 years	25 (1.9)	4 (2.7)	<0.01
41–50 years	146 (10.8)	3 (2.0)	
51–60 years	303 (22.5)	12 (8.1)	
61–70 years	550 (40.9)	41 (27.5)	
>70 years	322 (23.9)	89 (59.7)	
***Detection mode***			
Screening	937 (69.6)	55 (36.9)	<0.01
Clinical	405 (30.1)	94 (63.1)	
Missing	4 (0.3)	0 (0.0)	
***Side***			
Right	667 (49.6)	65 (43.6)	0.19
Left	679 (50.4)	84 (56.4)	
***T-Stage (Clin)***			
T1	1169 (86.8)	107 (71.8)	<0.01
T2	177 (13.2)	42 (28.2)	
***N-stage (Clin)***			
N0	1278 (94.9)	143 (96.0)	0.69
N+	68 (5.1)	6 (4.0)	
***Axillary surgery***			
SN	1091 (81.1)	109 (73.2)	<0.01
ALND	51 (3.8)	8 (5.4)	
SN + ALND	188 (14.0)	24 (16.1)	
Sampling	16 (1.2)	8 (5.4)	
***Invasive tumour size***			
0–10 mm	422 (31.4)	33 (22.1)	0.01
11–20 mm	695 (51.6)	78 (52.3)	
21–30 mm	229 (17.0)	38 (25.5)	
***Multifocality***			
No	1211 (90.0)	126 (84.6)	0.05
Yes	135 (10.0)	23 (15.4)	
***In situ component***			
Invasive only	651 (48.4)	76 (51.0)	0.55
Invasive + Cancer *in situ*	695 (51.6)	73 (49.0)	
***Extent of all tumour foci combined***			
1–20 mm	424 (31.5)	32 (21.5)	0.04
>20 mm	241 (17.9)	32(21.5)	
Missing	681 (50.6)	85 (57.0)	
***Histologic classification***			
Ductal	1097 (81.5)	121 (81.2)	0.97
Lobular	164 (12.2)	19 (12.8)	
Other	85 (6.3)	9 (6.0)	
***Grade***			
I	321 (23.8)	28 (18.8)	0.57
II	668 (49.6)	79 (53.0)	
III	345 (25.6)	41 (27.5)	
Missing	12 (0.9)	1 (0.7)	
***ER***			
Pos	1161 (86.3)	127 (85.2)	0.57
Neg	143 (10.6)	19 (12.8)	
Missing	42 (3.1)	3 (2.0)	
***PR***			
Pos	1050 (78.0)	115 (77.2)	0.64
Neg	253 (18.8)	31 (20.8)	
Missing	43 (3.2)	3 (2.0)	
***HER2***			
Neg	1179 (87.6)	135 (90.6)	0.53
Pos	136 (10.1)	12 (8.1)	
Missing	31 (2.3)	2 (1.3)	
***Ki67***			
Low	735 (54.6)	76 (51.0)	0.22
High	593 (44.1)	73 (49.0)	
Missing	18 (1.3)	0 (0.0)	
***Lymph node involvement***			
N0	1088 (80.8)	116 (77.9)	0.38
N+	258 (19.2)	33 (22.1)	

BCS = breast conserving surgery, Clin = Clinical, SN = Sentinel node biopsy, ALND = Axillary lymph node dissection, ER = Oestrogen receptor, PR = Progesterone receptor, HER2 = Human epidermal growth factor receptor 2.

There was no correlation between women opting for mastectomy between hospitals with or without radiation facilities (Table S3). When comparing women without medical contraindications for BCS and RT who chose a mastectomy to those who underwent BCS, age above 70 years, T-stage, and clinical detection were the strongest predictors for choosing mastectomy (Table 3).

## 4 Discussion

Women with BC ≤ 30 mm treated with mastectomy had, as expected, larger tumours compared to those treated with BCS. Other adverse tumour traits such as higher histological grade, positive lymph node status, and unfavourable tumour biology were also associated with a higher likelihood of undergoing mastectomy. Women reported to choose mastectomy by their own preference also had less favourable prognostic characteristics than women treated with BCS.

Several randomized trials have shown that survival is equivalent with either BCS combined with RT or mastectomy [[6], [7], [8]]. Numerous retrospective observational studies published in recent years suggest that BCS and RT offers superior survival compared to mastectomy [[1], [2], [3], [4], [5]], and it has been questioned whether mastectomy should at all be offered as an alternative when BCS and RT is feasible. However, in two propensity-score matched analyses, mastectomy was not associated with worse survival [11,12]. In the study by Landcasper et al. including more than 200.000 stage I-III matched patients, a large number of confounding variables were available and thery found no survival disadvantage for patients undergoing mastectomy compared to those undergoing lumpectomy [11]. This was corroborated by a more recent propensity score-matched analysis from Italy including 9170 patients and is consistent with the results of earlier randomized trials [12].

There is no unified theory why less extensive surgery should be protective for BC recurrence and lead to a better patient outcome. Proposed explanations is that the addition of RT provides biological effects that prevents early distant dissemination or that the whole-breast tangential field of radiation gives a protective effect in terms of axillary recurrence [3,9]. Over the last decades, local recurrence rates after BCS have fallen considerably and this along with improved contemporary RT has been another suggested explanation for the better survival outcomes [9,15]. Differences in outcome between BCS with RT and mastectomy might however be due to selection mechanisms. In most observational studies, adjustments for comorbidity or contraindications for radiotherapy is not available. Van Maaren et al. analyzed data from the Netherlands cancer registry between 2000 and 2004. In this study 10 year BC-specific survival was only improved for BCS + RT in T1N0 disease suggesting a confounding effect of comorbidities [4]. Whang et al. showed that women who underwent mastectomy had a substantially higher risk of death from heart and respiratory diseases within 3 years after diagnosis compared to women who underwent BCS, underlining the existing differences in comorbidity between treatment groups [1]. In the Swedish cohort study there was also a notable imbalance in the administration of adjuvant chemotherapy between women undergoing BCS compared to mastectomy and almost 20 % of the women undergoing mastectomy who had axillary lymphnode metastases did not receive regional RT contrary to Swedish national guidelines [3]. In the other study from Sweden they were able to adjust both for tumor characteristics, socioeconomic status and comorbidities but the analyses were not adjusted for adjuvant systemic treatments [5]. There may also be additional confounders not noted in this or other studies such as data on family history of BC or presence of genetic mutations.

The total extent of all tumours in case of multifocal or multicentric BC is rarely reported.

Multifocality was far more common in the mastectomy group in the present study. This was also true in the comparison with women who opted to undergo mastectomy by their own choice. The AJCC TNM staging system does not account for multifocality, nor the total extent of all tumour foci combined. Multifocality is associated with decreased survival and the total tumour burden may be grossly underestimated in cases of multifocality [16,17]. Multifocality is not always known at diagnosis but found in the postoperative specimen, thus potentially leading to a re-excision or finally a mastectomy. Indeed, in the present cohort more than half of the women who underwent mastectomy after initial BCS had multifocal tumour foci.

As expected, treatment decision was for most patients (two out of three), reported to be based on medical reasons. This is also in keeping with national guidelines that recommendations for surgery in the surgical and oncological consultation should be based primarily on medical, rational grounds. Most commonly, the decision was correlated to clinical tumour stage, which is in accordance with other studies [18]. Treatment choice beyond disease stage is also influenced by genetic and lifestyle related factors, as well as comorbidities, that may or may not be included in registers. A limitation of the present study is the lack of information on hereditary factors in the register and thus the potential effect of increased familiar risk could unfortunately not be analyzed here.

Increasing age was associated with higher likelihood of mastectomy, which is in line with a systematic review including 25 trials by Gu et al. [18]. The difference seen in axillary surgery between women opting for mastectomy and women selected for BCS, can at least to some extent indicate that these patients were not considered candidates for chemotherapy, which is further strengthened by the present findings that women above 70 considerably more often receive mastectomy by their own choice.

In contrast to most other studies, no correlation was found between the patient's preference of a mastectomy and geographical distance to RT facility. In the review by Gu et al., five out of seven studies showed higher mastectomy rates with increased distance to RT facility, while two showed no difference [18]. Factors that have been reported to influence the choice of mastectomy are socioeconomic status [19] and personal beliefs such as fear of BC recurrence or concern about side-effects of RT among others [13,18,20,21]. Information regarding socioeconomic status and personal beliefs were not available in the NBCR and were thus not possible to account for in the present analysis.

The strength of this study is the population-based setting and that the medical data and the reason for choosing mastectomy were registered prospectively, thus not sensitive to recall bias. The proportion of BCS performed in the Northern Region was comparable to, and even slightly higher than the average proportion in Sweden, indicating that the study cohort is representative for Swedish BC. The NBCR has a documented high coverage and validity [14], and variables not usually available such as presence of multiple tumour foci and widespread DCIS were also included.

According to the register, one-third of the mastectomies were performed by the patient's own choice. A limitation is that it remains unclear whether this was influenced by hereditary factors, socioeconomic factors, personal beliefs, or the treating physician's attitude. Another limitation is that the staging system has broad categories; e.g. the T2 and N1 categories include a broad range of tumours with significantly different outcomes. Thus, a more detailed tumour categorization may have revealed even larger differences in the distribution of tumours with different prognosis.

## 5 Conclusion

To conclude, women with invasive BC ≤ 30 mm undergoing mastectomy have less favourable prognostic characteristics than those treated with BCS. This is also true for women reported to have chosen mastectomy of own preference. Our study show that choice of surgery is strongly associated with factors that are important drivers in the difference in prognosis between women undergoing BCS with RT compared to mastectomy. Failure to adjust for these factors may bias results in outcome studies in favour of BCS.

## Funding

This study was funded by research grants from the Department of Research and Development, Vasternorrland County Council (to E.S.), Swedish Breast Cancer Society (to M.S.), the Percy Falk Foundation (to M.S.), and Visare Norr (grant number 68146 to C.W.)

## Ethical approval

The study was performed after ethical approval by the Swedish Ethical Review Board (EPM Dnr:2019–04916), and in accordance with the principles of the Helsinki Declaration.

## CRediT authorship contribution statement

**Emma Söderberg:** Conceptualization, Formal analysis, Investigation, Methodology, Project administration, Validation, Visualization, Writing - original draft, Writing - review & editing. **Fredrik Wärnberg:** Conceptualization, Investigation, Methodology, Supervision, Visualization, Writing - review & editing. **Anna-Karin Wennstig:** Conceptualization, Methodology, Supervision, Writing - review & editing. **Greger Nilsson:** Conceptualization, Methodology, Visualization, Writing - review & editing. **Hans Garmo:** Data curation, Formal analysis, Investigation, Methodology, Writing - review & editing. **Lars Holmberg:** Conceptualization, Methodology, Project administration, Supervision, Writing - review & editing. **Carl Blomqvist:** Conceptualization, Methodology, Writing - review & editing. **Malin Sund:** Conceptualization, Funding acquisition, Methodology, Project administration, Resources, Supervision, Writing - review & editing. **Charlotta Wadsten:** Conceptualization, Data curation, Formal analysis, Funding acquisition, Investigation, Methodology, Project administration, Software, Supervision, Validation, Visualization, Writing - review & editing.

## Declaration of competing interest

None.
