# Supplementary material for: Association of clinicopathologic variables and patient preference with the choice of surgical treatment for early-stage breast cancer: A registry-based study
Source: Breast. 2023 Nov 30;73:103614. doi: 10.1016/j.breast.2023.103614 (PMC10746552; doi:10.1016/j.breast.2023.103614)
Supplement: Multimedia component 1 [file mmc1.docx]

**Supplementary Figures and Tables**

Table S1. Histopathological features by type of surgery

|  | All  n=1860 | BCS  n=1346 | Mastectomy  n=514 | Upfront mastectomy  n=452 | Mastectomy after initial BCS  n=62 |
| --- | --- | --- | --- | --- | --- |
| **Largest invasive tumour** |  |  |  |  |  |
| < 10 mm | 537 (28.9) | 422 (31.4) | 115 (22.4) | 93(20.6) | 22 (35.5) |
| 11-20 mm | 928 (49.9 | 695 (51.6) | 233 (45.3) | 211 (46.7) | 22 (35.5) |
| 21-30 mm | 395(21.2) | 229 (17.0) | 166 (32.3) | 148 (32.7) | 18 (29.0) |
| **Focality** |  |  |  |  |  |
| Unifocal | 1551 (83.4) | 1211 (90.0) | 340 (66.1) | 312 (69.0) | 28 (45.2) |
| Multifocal | 309 (16.6) | 135 (10.0) | 174 (33.9) | 140 (31.0) | 34 (54.8) |
| **Presence of in situ component** |  |  |  |  |  |
| Invasive only | 837 (45.0) | 651 (48.4) | 186 (36.2) | 179 (39.6) | 7 (11.3) |
| Invasive+in situ | 1023 (55.0) | 695 (51.6) | 328 (63.8) | 273 (60.4) | 55 (88.7) |
| **Extent of all foci combined*** |  |  |  |  |  |
| < 10 mm | 147 (16.1) | 140 (21.1) | 7 (2.8) | 7 (3.2) | 0 (0.0) |
| 11-20 mm | 353 (38.6) | 284 (42.7) | 69 (27.7) | 67 (30.3) | 2 (7.1) |
| 21-30 mm | 222 (24.3) | 151 (22.7) | 71 (28.5) | 65 (29.4) | 6 (21.4) |
| 31-40 mm | 73 (8.0) | 45 (6.8) | 28 (11.2) | 24 (10.9) | 4 (14.3) |
| 41+ mm | 119 (13.0) | 45 (6.8) | 74 (29.7) | 58 (26.2) | 16 (57.1) |
| **Histological type** |  |  |  |  |  |
| Ductal | 1491 (80.2) | 1097 (81.5) | 394 (76.7) | 350 (77.4) | 44 (71.0) |
| Lobular | 256 (13.8) | 164 (12.2) | 92 (17.9) | 75 (16.6) | 17 (27.4) |
| Other | 113 (6.1) | 85 (6.3) | 28 (5.4) | 27 (6.0) | 1 (1.6) |
| **Grade** |  |  |  |  |  |
| Grade I | 389 (20.9) | 321 (23.8) | 68 (13.2) | 60 (13.3) | 8 (12.9) |
| Grade II | 940 (50.5) | 668 (49.6) | 272 (52.9) | 239 (52.9) | 33 (53.2) |
| Grade III | 511 (27.5) | 345 (25.6) | 166 (32.3) | 145 (32.1) | 21 (33.9) |
| Missing | 20 (1.1) | 12 (0.9) | 8 (1.6) | 8 (1.8) | 0 (0.0) |
| **ER status** |  |  |  |  |  |
| ER pos | 1586 (85.3) | 1161 (86.3) | 425 (82.7) | 372 (82.3) | 53 (85.5) |
| ER neg | 220 (11.8) | 143 (10.6) | 77 (15.0) | 69 (15.3) | 8 (12.9) |
| Missing | 54 (2.9) | 42 (3.1) | 12 (2.3) | 11 (2.4) | 1 (1.6) |
| **PR status** |  |  |  |  |  |
| PR pos | 1428 (76.8) | 1050 (78.0) | 378 (73.5) | 332 (73.5) | 46 (74.2) |
| PR neg | 377 (20.3) | 253 (18.8) | 124 (24.1) | 109 (24.1) | 15 (24.2) |
| Missing | 55 (3.0) | 43 (3.2) | 12 (2.3) | 11 (2.4) | 1 (1.6) |
| **HER2 status** |  |  |  |  |  |
| HER2 neg | 1605 (86.3) | 1179 (87.6) | 426 (82.9) | 374 (82.7) | 52 (83.9) |
| HER2 pos | 215 (11.6) | 136 (10.1) | 79 (15.4) | 71 (15.7) | 8 (12.9) |
| Missing | 40 (2.2) | 31 (2.3) | 9 (1.8) | 7 (1.5) | 2 (3.2) |
| **Ki67** |  |  |  |  |  |
| Ki67 low | 979 (52.6) | 735 (54.6) | 244 (47.5) | 218 (48.2) | 26 (41.9) |
| Ki67 high | 856 (46.0) | 593 (44.1) | 263 (51.2) | 227 (50.2) | 36 (58.1) |
| Missing | 25 (1.3) | 18 (1.3) | 7 (1.4) | 7 (1.5) | 0 (0.0) |
| **N status** |  |  |  |  |  |
| pN0 | 1446 (77.7) | 1088 (80.8) | 358 (69.6) | 316 (69.9) | 42 (67.7) |
| pN+ (1-4) | 414 (22.3) | 258 (19.2) | 156 (30.4) | 136 (30.1) | 20 (32.2) |

*Data available in 2015-2016

BCS = breast conserving surgery, ER = Oestrogen receptor, PR = Progesterone receptor, HER2 = Human epidermal growth factor receptor 2, N status = Nodal status

Table S2. Comparison of clinical characteristics for women with or without registered reason for mastectomy

| Variable | Reason registered  n=431 | Reason not registered  n=83 | p-value |
| --- | --- | --- | --- |
| Age (mean) | 65.9 years | 67.4 years | 0.39 |
| Screening detected | 37.8 % | 25.3 % | 0.03 |
| T1  T2 | 66.1 %  33.9 % | 54.2 %  45.8 % | 0.11 |
| N0  N+ (1-4 metastases) | 90.7 %  9.3 % | 88.0 %  12.0 % | 0.44 |

Table S3. Patients’ choice of mastectomy in hospitals with or without radiotherapy facility

|  | Hospital with RT facility | Hospital without RT facility | p-value |
| --- | --- | --- | --- |
| Overall mastectomy rate  (%) | 244/915 (26.7 %) | 270/945 (28.6 %) | 0.36 |
| Mastectomies performed based on the patient’s own choice (%) | 75/915 (8.2 %) | 83/945 (8.8 %) | 0.65 |

RT = Radiotherapy
